# Networks and Graphs Discovery in Metabolomics Data Analysis and Interpretation

**DOI:** 10.3389/fmolb.2022.841373

**Published:** 2022-03-08

**Authors:** Adam Amara, Clément Frainay, Fabien Jourdan, Thomas Naake, Steffen Neumann, Elva María Novoa-del-Toro, Reza M Salek, Liesa Salzer, Sarah Scharfenberg, Michael Witting

**Affiliations:** ^1^ Section of Nutrition and Metabolism, International Agency for Research on Cancer (IARC-WHO), Lyon, France; ^2^ Toxalim (Research Centre in Food Toxicology), Université de Toulouse, INRAE, ENVT, INP-Purpan, UPS, Toulouse, France; ^3^ MetaboHUB-Metatoul, National Infrastructure of Metabolomics and Fluxomics, Toulouse, France; ^4^ European Molecular Biology Laboratory (EMBL), Genome Biology Unit, Heidelberg, Germany; ^5^ Bioinformatics and Scientific Data, Leibniz Institute of Plant Biochemistry, Halle (Saale), Germany; ^6^ German Centre for Integrative Biodiversity Research (iDiv) Halle-Jena-Leipzig, Leipzig, Germany; ^7^ Bruker BioSpin GmbH, Ettlingen, Germany; ^8^ Research Unit Analytical BioGeoChemistry, Helmholtz Zentrum München, Neuherberg, Germany; ^9^ Metabolomics and Proteomics Core, Helmholtz Zentrum München, Neuherberg, Germany; ^10^ Chair of Analytical Food Chemistry, TUM School of Life Sciences, Freising, Germany

**Keywords:** metabolic network, untargeted metabolomics, graph-based analysis, knowledge network, experimental network, metabolism, systems biology

## Abstract

Both targeted and untargeted mass spectrometry-based metabolomics approaches are used to understand the metabolic processes taking place in various organisms, from prokaryotes, plants, fungi to animals and humans. Untargeted approaches allow to detect as many metabolites as possible at once, identify unexpected metabolic changes, and characterize novel metabolites in biological samples. However, the identification of metabolites and the biological interpretation of such large and complex datasets remain challenging. One approach to address these challenges is considering that metabolites are connected through informative relationships. Such relationships can be formalized as networks, where the nodes correspond to the metabolites or features (when there is no or only partial identification), and edges connect nodes if the corresponding metabolites are related. Several networks can be built from a single dataset (or a list of metabolites), where each network represents different relationships, such as statistical (correlated metabolites), biochemical (known or putative substrates and products of reactions), or chemical (structural similarities, ontological relations). Once these networks are built, they can subsequently be mined using algorithms from network (or graph) theory to gain insights into metabolism. For instance, we can connect metabolites based on prior knowledge on enzymatic reactions, then provide suggestions for potential metabolite identifications, or detect clusters of co-regulated metabolites. In this review, we first aim at settling a nomenclature and formalism to avoid confusion when referring to different networks used in the field of metabolomics. Then, we present the state of the art of network-based methods for mass spectrometry-based metabolomics data analysis, as well as future developments expected in this area. We cover the use of networks applications using biochemical reactions, mass spectrometry features, chemical structural similarities, and correlations between metabolites. We also describe the application of knowledge networks such as metabolic reaction networks. Finally, we discuss the possibility of combining different networks to analyze and interpret them simultaneously.

## Introduction

Metabolomics research is based on various opportunities to uncover the metabolites contained in biological samples. To characterize and quantify metabolites in biological samples, different types of metabolite separation techniques - such as Liquid Chromatography (LC), Gas Chromatography (GC), Capillary Electrophoresis (CE), or Ion Mobility (IM)–are coupled to a Mass-Spectrometry (MS) system. High-performance mass spectrometry systems generate increasingly complex datasets. Two major approaches are used in metabolomics: targeted methods look for a pre-selected list (or class) of metabolites, and untargeted metabolomics covers as many metabolites as possible (Schrimpe-Rutledge et al., 2016). However, in untargeted metabolomics research, processing, analyzing, and interpreting the complex datasets that are generated are major challenges. Nuclear Magnetic Resonance (NMR) techniques are also used in metabolomics (Emwas et al., 2019), but most of the network and graph methods covered here are rather focused on MS-based metabolomics. As multiple network constructions approaches presented here are relying on the specificity of data generated by MS (e.g., fragmentation or adducts).

The analysis of untargeted metabolomics datasets is frequently limited by the ability to annotate and identify metabolites at a large scale (hundreds or thousands of metabolites). Data interpretation is often reductionist and limited to a few specific metabolic processes or metabolites, found to be statistically significantly associated with a phenotype of interest. This implies that a potentially large part of the detected metabolites will be ignored if they appear not statistically significant to the question at hand. Importantly, the recent use of network and graph-based methods to analyze metabolomics data opened the possibility of metabolomics data systematic analysis (Kell and Goodacre, 2014; Perez De Souza et al., 2020).

**FIGURE 1:**
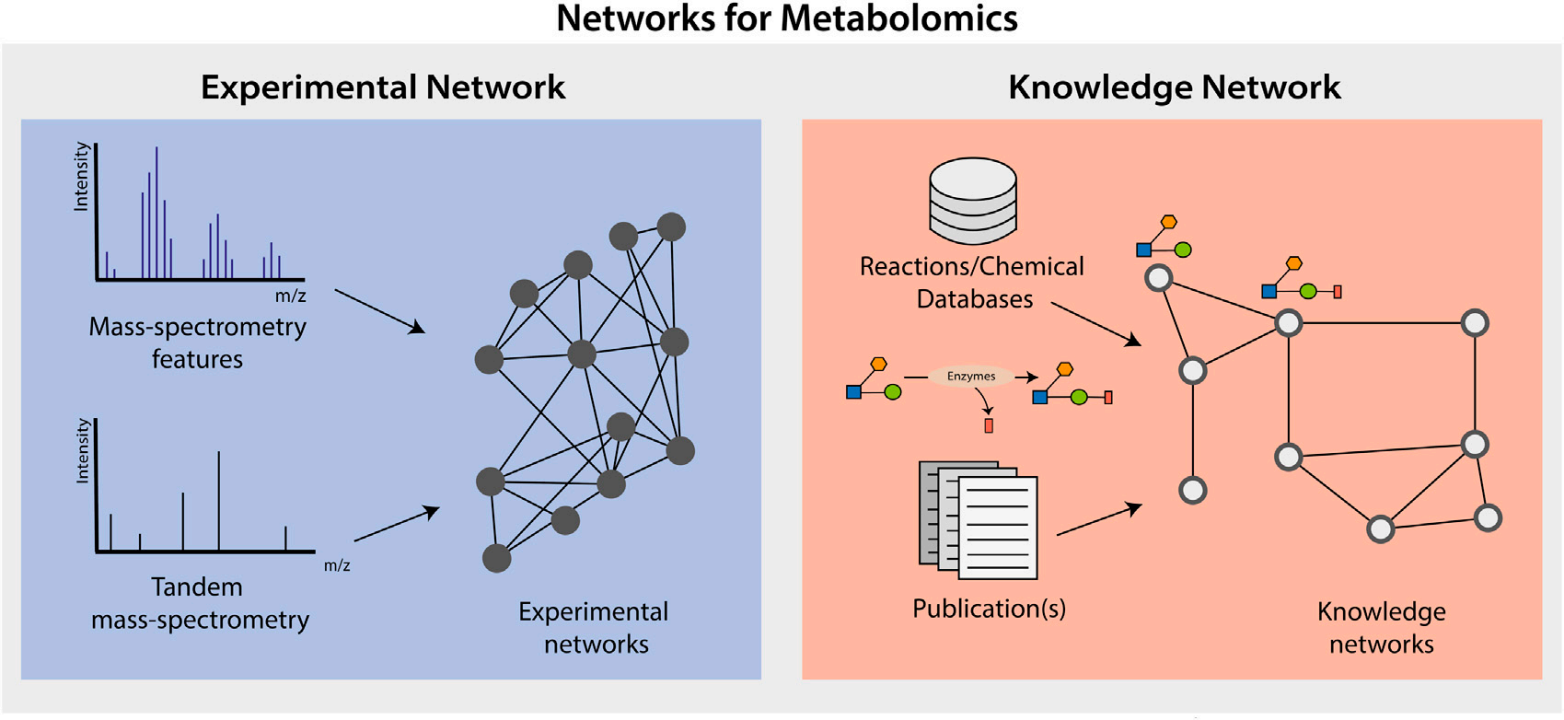
Graphical Abstract. In this review we will be presenting two major types of networks and graphs used to analyze and interpret metabolomics data, knowledge networks and experimental networks.

There are two major types of networks used with metabolomics data: knowledge and experimental (Figure 1). Knowledge networks are generated from biochemical or biological knowledge and allow interpreting metabolomics data in the context of prior biological knowledge, such as metabolic pathways and enzymatic reactions. For instance, a metabolic network is a knowledge network, where metabolites and their known biochemical conversions are represented as nodes and edges, respectively. On the other hand, experimental networks are generated from the metabolomics data itself, based on relationships between possible or identified metabolites in the data (e.g., spectral similarity, or correlation). Notably, both types of networks (i.e., knowledge and experimental) can be used with advanced statistical methods, graph analysis, and data analysis approaches to study the interconnected data.

The words “network” and “graph” are often used interchangeably, and preferred terms depend on fields and traditions. We will refer to the curated lists of biochemical reactions and their participants (e.g., substrate, products, enzymes, and genes) as “metabolic networks” (following current usage). We will refer as “metabolic graphs” the different entity-relationship structures that can be derived from such biochemical reaction lists to perform topological analysis (such as compound graphs and reaction graph), to avoid the ambiguity with their source material.

Metabolism consists of enzymatic and non-enzymatic reactions converting metabolites to produce energy (catabolism), build up biomass (anabolism), or respond to external stimuli. Metabolism is often seen as functional modules conserved across organisms. Examples of such functional modules are the central carbon metabolism, which is highly conserved, and the secondary (AKA specialized) metabolism, which differs vastly among organisms. Furthermore, co-metabolism in communities (such as microbiomes) increases metabolic capacities and leads to a very high diversity of metabolites. In this context, the unilateral interpretation of metabolomics data may hide complex systemic changes spanning across several pathways. This is especially the case with metabolic chart representations that are designed to focus on knowledge-based biochemical pathways and ignore the interconnections between pathways. Additionally, the lack of consensus on the partitioning of metabolic pathways or modules from one database to another can lead to major discrepancies in the analysis (Stobbe et al., 2012; Altman et al., 2013). Instead, it is possible to represent the metabolism as a network of metabolites connected by specific or promiscuous enzymatic and non-enzymatic reactions. Importantly, in such a network, we can also represent interconnections between metabolites which may look unrelated but that are connected via different pathways. Genome-Scale Metabolic Networks (GSMNs) are designed to represent this information based on genomics knowledge, providing a systemic view of the metabolism. Nevertheless, GSMNs are based on metabolism knowledge coming from genome annotation, which prevents the integration of many metabolites since there are gaps in knowledge (e.g., secondary metabolism) (Frainay et al., 2018). These gaps require us to expand those metabolic networks using experimental data from metabolomics experiments.

Untargeted MS data, either based on direct infusion or coupled to different types of separation techniques (e.g., LC, GC, CE, or IM), is characterized by features for which we measured the mass-to-charge ratio (m/z value with a mass accuracy of just a few ppm, depending on the instrumentation), the abundance (either a peak intensity or a peak area), an additional separation index (retention or migration time, mobility, or collisional cross-section value), and the associated fragmentation pattern, if collected. Based on these data, metabolites can be annotated or identified with different confidence levels, according to the Metabolomics Standard Initiative (MSI) (Fiehn et al., 2007; Sumner et al., 2007; Schymanski et al., 2014). The highest level of confidence (i.e., level 1) is achieved by a matching in at least two independent and orthogonal data (e.g., mass spectrum and retention time/index) between the metabolite feature and its authentic reference standard, both of which must be analyzed under identical conditions. This identification level is often only possible for metabolites for which reference standards are available in the respective laboratory. Indeed, recent work has shown that only a small part of the metabolites found in metabolic networks of different organisms is covered by at least one reference spectrum (Frainay et al., 2018). Lower-confidence annotations (i.e., levels 2 and 3) can be achieved by matching the metabolite feature with spectral libraries or using in-silico tools, such as MetFrag (Ruttkies et al., 2019) or CSI:FingerID (Dührkop et al., 2015), among others (Misra and van der Hooft, 2016; Spicer et al., 2017; Misra, 2021). Assessing the structural similarity relationship via spectral similarity has proven to be a powerful tool to guide annotation of unknown metabolites (Wang et al., 2016), since chances of having structurally homologous metabolites detected in parallel are high. However, metabolites are generally not detected as isolated entities, but as part of larger sets of metabolites of the same chemical classes.

Here, we will describe the current state of the art in terms of networks and graphs usage for metabolomics, detailing their characteristics and applications. We will first focus on experimental networks (such as those based on mass differences, adducts and features, structure similarities, and correlation), which are generated from metabolomics data. Notably, experimental networks have been used to annotate and identify metabolites (Loos and Singer, 2017; Schmid et al., 2021), as well as to better understand biochemical relationships between metabolites (Schollée et al., 2017; Naake and Fernie, 2019). We will also describe knowledge networks (such as ontology-based networks (Dührkop et al., 2020) and GSMNs), which are increasingly used to interpret metabolomics data (Kell and Goodacre, 2014; Frainay and Jourdan, 2017) and to annotate metabolites (Silva et al., 2014; Schmid et al., 2021). While each network (experimental or knowledge-based) covers a specific aspect of the studied biology, there are benefits in integrating them. For instance, experimental networks can help in filling the gaps in current knowledge-based networks by mapping the nodes in the knowledge-based network (i.e., metabolites) with the corresponding nodes in the experimental networks (i.e., features) and identifying missing metabolites. Importantly, knowledge-based networks provide a biological context to help interpret and analyze experimental networks. To emphasize this, we finish this review by presenting combined networks analysis approaches, such as multi-layer networks applied to the field of metabolomics.

## Experimental Networks

Experimental networks are directly derived from the acquired untargeted metabolomics data. Depending on the type of network, either MS^1^, MS^2^, or MS^n^ data is used. Each network tackles a different aspect of the compounds “metabolic relatedness”, with specific assumptions and shortcomings, which we will describe in the following sections. We will discuss how mass differences, adducts and features, structure similarities and correlation data can be used to build different experimental networks.

**FIGURE 2:**
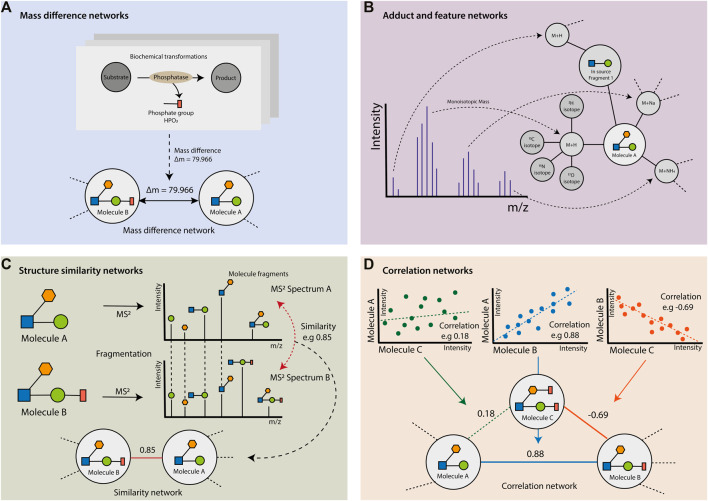
Metabolomics-based experimental networks. **(A)** Mass difference networks: the biochemical transformations entail gains and/or losses of atoms that lead to changes in the metabolites’ molecular formula and, therefore, changes in the exact mass of molecules connected via a reaction. Here, the biochemical transformation by a phosphatase causes the loss of a phosphate group (HPO3), leading to a mass difference of 79.966 between the substrate metabolite (Molecule **(B)** and the product metabolite (Molecule A). **(B)** Adduct and feature networks: metabolites have multiple possible adducts and features associated with them. Each detected adduct, isotopologue, and ion-source fragments can be represented as nodes. Adducts (e.g., M + H) are connected to corresponding or potential metabolites. Similarly, the isotopologues of an adduct are linked to the associated adduct nodes (e.g., 13C isotopologue of M + H). Finally, ion-source fragments (here in-source fragment 1) with their associated adducts and isotopologues can be linked to the corresponding node metabolite. **(C)** Structure similarity networks: the structural similarity between metabolites detected by MS methods can be observed and calculated based on their MS/MS spectra. The fragmentation patterns will be similar for two metabolites with a shared core structure (represented as circles, squares, and polygons), but a difference due to a chemical reaction (i.e., the residue represented by the red rectangle). The calculated similarity (i.e., 0.85) between two MS2 spectra is the weight of the edge linking the corresponding metabolite pair. **(D)** Correlation networks: the correlation between the abundances of two metabolites can be calculated and used as a weight for the edge (i.e., 0.88 or −0.69) between two metabolites’ node (i.e., between molecules A and B, or between molecules B and C). The correlation levels considered as non-significant (i.e., 0.18) can be ignored and excluded from the correlation network (i.e., the edge between molecules A and C).

It is important to highlight that experimental networks complement each other to decipher the metabolic relationships between compounds. As two faces of the same coin, spectral similarity networks can suggest substrate-product links from expected global chemical similarity (Figure 2C); while mass difference networks represent the substrate-product links from characteristic differences due to local chemical structure changes (Figure 2A). Extra evidence of the existence of such substrate-product links can come from correlation networks, which reveal possible causal relationships between the changes in the metabolites’ abundances (Figure 2D). Finally, the adduct and feature networks can increase the confidence in metabolites’ annotations in the networks, based on characteristic patterns, associated to individual compounds in mass spectrometry (Figure 2B).

### Mass Difference Networks

The biochemical transformations are characterized by the gain or loss of atoms, which lead to changes in the metabolites’ molecular formula and, therefore, variations in the exact mass of pairs of molecules connected by a reaction. These changes can be measured in MS-based metabolomics as differences between pairs of m/z values (Figure 2A) to generate a mass difference network (Table 1).

**TABLE 1:** Description of the key characteristics of mass difference networks.

Mass difference networks’ main characteristics	
Nodes	Features, low level annotations (m/z + RT)
Edges	Putative substrate-product relationships from biochemical transformations’ characteristic patterns
Main Hypothesis	Many biochemical reactions involve functional group transfer, yielding a characteristic mass shift. Feature pairs with mass differences matching those patterns might be involved in a reaction transferring the corresponding group
Limitations	The mass differences between a pair of features may correspond to an existing reaction, but it can happen that these two features do not correspond to a real biotransformation between the corresponding metabolites, leading to spurious edges

The mass difference approach can be used with known biotransformations and their corresponding mass differences to find potential biochemical reactions explaining the difference between m/z values (Breitling et al., 2006; Tziotis et al., 2011). Therefore, in a mass difference network, the features with their corresponding m/z values are represented as nodes, and the mass differences between pairs of m/z values that match a pre-defined transformation as edges (Figure 2A). Potential transformations can be derived from metabolic reaction databases, such as KEGG, MetaNetX, MetExplore, etc. (Jeffryes et al., 2015; Hadadi et al., 2016; Kanehisa et al., 2017; Cottret et al., 2018; Ebastien Moretti et al., 2021). If seed formulae (e.g., from identified metabolites) are available, information on known biochemical transformations can also be used to calculate molecular formulae, by propagating the difference formulae within the network. By comparing the frequency of certain mass differences between different conditions, conclusions on potential biochemical responses can be drawn (Moritz et al., 2016). However, this approach requires a priori hypothesis on data to generate an appropriate transformation list. Notably, features connected by a mass difference that is not included in the transformation list will not be connected in the mass difference network. Moreover, if metabolites from a reaction series are not detected by the instrument, there will be gaps (missing nodes) in the reconstructed network. For certain instances, this can be overcome, e.g., by combining several mass differences into one corresponding to multiple biotransformations. For example, gaps in the network for series of alkyl chains (C_n_H_2n+1_) can be filled by adding C_2_H_4_ to the transformation list to cover for two times CH_2_ or by adding C_4_H_8_ to cover two times C_2_H_4_.

Another approach frequently used is to include all mass differences between all pairs of features, to generate mass difference networks. The result is a fully connected graph where all features are connected to each other, and their edges represent their mass differences. It is challenging to find meaningful network motifs in such a graph, since even non-biochemically related features would still be connected by an edge, with the sole purpose of holding the mass difference attribute. One solution to reduce irrelevant links is to filter out edges connecting features with low intensity/concentration correlation. It is also possible to filter edges following a specific Retention Time (RT) trend. For example, there is a predictable RT and mass difference between products and substrates of a specific reaction, which can be propagated from a known metabolite in the network to neighboring metabolites. This approach can result in the discovery of new biochemical transformations unbiased, as it does not use biotransformation-based mass differences (Morreel et al., 2014). However, the interpretation of the results might become complicated, as it represents a combination of several losses and gains of atoms. As an example, in the transamination reaction, transamination of pyruvate (C_3_H_4_O_3_ to alanine (C_3_H_7_NO_2_ is accompanied by the gain of one nitrogen and three hydrogens (NH_3_ = 17.03) and the loss of one oxygen (O = 15.99), yielding to a net mass difference of 1.0316, from which no meaningful formula can be calculated.

There are different tools for the generation of mass difference networks. The tool mzGroupAnalyzer can generate a mass difference network based on an input list of transformations atom differences, it allows visualization of the metabolites elements composition with a van Krevelen diagram (based on H/C and O/C ratios) to identify patterns of structural similarity between compounds (Doerfler et al., 2014). MetaNetter is a Cytoscape plugin that performs ab initio prediction of mass difference networks from high-resolution data, such as Orbitrap or Fourier transform ion cyclotron resonance mass spectrometer (FT-ICR-MS) (Jourdan et al., 2008; Burgess et al., 2017). MetNet is an R package that represents one of the most prominent tools to generate mass difference networks based on pre-defined transformations lists; in combination with other types of information (such as RT shifts or correlations) (Naake and Fernie, 2019). The inclusion of such additional information reduces the connection degree between features, as it constrains the creation of edges between nodes with a threshold of correlations and/or with specific RT shifts.

### Adducts and Features Networks

Mass differences do not only occur due to biological transformations between metabolites, but might also appear due to different physicochemical effects when introducing the metabolites to the MS. These “non-biological” mass differences can be represented in adducts and feature networks (Table 2). The relationships between features are used for grouping and deconvoluting the detected m/z signals, as in the R package CAMERA (Kuhl et al., 2012). Analysis of mass differences is greatly enhanced using chromatographic separation, as the RT windows help to separate metabolites features. Isotopes, adducts, as well as in-source fragments of the same metabolite show (theoretically perfect) co-elution. A particular example of co-elution is the annotation of [M + H]^+^ and [M-H_2_O + H]^+^, while [M-H_2_O + H]^+^ normally co-elutes with [M + H]^+^, metabolites that differ in H_2_O in their formulas have different chemical structures and therefore different RTs.

**TABLE 2:** Description of the key characteristics of adducts and features networks.

Adducts and features networks’ main characteristics	
Nodes	Features, intermediate level annotations (m/z + RT + adduct information)
Edges	Putative relationships between features, such as adducts of a metabolite, in-source fragments of a metabolite, or isotopologues of an adduct
Main Hypothesis	Same as mass difference networks, but with more detailed description using RT separation and characteristic patterns associated to adducts ions formation, ion-source fragmentations, and isotope patterns
Limitations	The mass differences between two features can correspond to a chemical relationship between two features (e.g., isotopologues or adducts), but these features correspond to the same metabolite

In-source fragmentation (ISF) is a common phenomenon that occurs in Electrospray ionization (ESI). ISF is the dissociation of a molecule that occurs within the ionization source of the mass spectrometer. During ESI, molecules gain additional internal energy that is released, resulting in the fragmentation of the molecule. This fragmentation generates additional precursor ions that can lead to false positive annotations of molecular features (Gathungu et al., 2018). There are several tools that can help with the identification of ISF of the same metabolite, e.g., CliqueMS, an R package that groups co-eluting features, based on similarity networks (Senan et al., 2019). Another recently developed R package that recognizes in-source fragments is ISFrag. ISFrag checks for co-elution, presence of the in-source fragment in the precursor MS^2^ spectra, and spectral similarity (Guo et al., 2021).

Ion identity networking is used to generate a network based on the relationships between ion species linked to the same compound as well as structurally similar compounds, which enhances compound annotation (Nothias et al., 2020). The detected ion-source fragments and their associated adducts and isotopologues can be represented in the network as nodes with edges linking them to their associated metabolite nodes (Figure 2B).

Certain mass differences might be found in a consecutive manner, e.g., CH_2_ or C_2_H_4_ for a homologous series, through an increase in an acyl chain length. Longer acyl chains lead to a higher RT in Reverse-Phase (RP) chromatography. Loos and Singer developed functionalities for the identification of homologous series by detecting series of mass differences following a given RT trend (Loos and Singer, 2017).

### Structure Similarity Networks

Typically, molecules connected via biochemical reactions are chemically similar since they often share common substructures. This resemblance can be expressed by chemical similarity measures, such as the Tanimoto similarity (Bender and Glen, 2004; Bajusz et al., 2015). It is important to note that similarity measures can only be calculated between identified compounds, since they require chemical structures as input (Table 3).

**TABLE 3:** Description of the key characteristics of structure similarity and MS/MS networks.

Structure similarity networks’ main characteristics	
Nodes	Features, high level annotations (m/z + RT + MS^2^)
Edges	Calculated similarity between two MS/MS spectra of two fragmented adducts
Main Hypothesis	Reactions tend to involve substrate-products pairs with high structural similarity. Thus, detected compounds with high structural similarity might be substrate/product of the same reaction
Limitations	Many compounds with structural similarity are not involved in the same reactions or pathways, which yields to false positives
	Depending on the methods and instruments, not every feature corresponding to a metabolite will be fragmented

In untargeted metabolomics, the MS^2^ fragmentation data is mostly generated using Data-Dependent Acquisition (DDA), which results in the fragmentation of the most abundant features. Fragmentation data can be used to infer (to a certain degree) structural similarity. Consequently, chemically similar compounds are likely to show at least partially similar fragmentation patterns. Note that the spectral differences can be both varying fragment masses and neutral loss differences. Molecules that have a shared core structure (e.g., an aglycon) can have differences due to the chemical reaction (e.g., additions of glycosyl groups) linked by the similarity of their MS^2^ spectra. Additionally, metabolites within the same compound class also show similar fragmentation patterns, even if they are not connected via biochemical reactions. An example is the fragmentation of glycerolipids, such as di- and tri-acylglycerols, which show characteristic neutral losses of fatty acid chains (Murphy et al., 2007).

Spectral similarity networks connect MS^2^ spectra of features or metabolites that show spectral similarity values above a certain threshold (Figure 2C). Therefore, finding metabolites within the same compound class or a similar one connected by biochemical reactions.

Different algorithms have been developed to use spectral similarity (based on different metrics, such as cosine or modified cosine similarities) to construct molecular similarity networks, as a proxy for structural similarity (Demuth et al., 2004; Aguilar-Mogas et al., 2017). The first application of molecular similarity networks was proposed by Watrous et al. Their similarity measure was based on a modified cosine score, which considers the mass difference between precursor masses. The mass differences between the precursor masses are applied to the fragments in the MS^2^ spectra, leading to a match of fragment peaks, either directly within a specific mass error or matching the mass plus the differences of the precursor masses. However, such spectral similarity networking only works on MS^2^ spectra and merges all spectra from the same precursor m/z, ignoring the fact that different isomers might elute at different RTs (Watrous et al., 2012).

In DDA, the intensities of the fragments are often not representative of a feature abundance in different samples since the measurement of an MS^n^ spectrum is, in most cases, not triggered at the apex of a chromatographic peak. Feature-based molecular networking uses the abundance of the MS^1^ feature (peak area or intensity), its RT, and the corresponding MS^2^ spectra as input and therefore allows the differentiation of isomeric structures based on chromatography (Nothias et al., 2020). In the resulting networks, abundances can be used as an added criterion for data analysis, revealing potential biological links. However, in such networks, different adducts from a single compound might end up in separated sub-networks, based on highly similar fragmentation of adducts. Ion identity networking has been introduced to combine these sub-networks to group those adducts by combining molecular networking and MS^1^ adduct detection algorithms, such as feature grouping and shape correlation (Schmid et al., 2021). This approach can also incorporate features into the network that have been identified as adducts but lack MS^2^ information.

The most prominent tool-set used for molecular networking has been developed by the Global Natural Product Social Molecular Networking (GNPS; http://gnps.ucsd.edu) community (Wang et al., 2016). GNPS is an open-access platform that allows storing and analyzing MS^2^ data, including molecular network generation using a modified cosine score and spectral library matching, followed by possible online visualizations.

Another example for generating molecular networks based on spectral similarity is MetGem, which utilizes the t-distributed stochastic neighbor embedding (t-SNE) algorithm to visualize the cosine scores calculated in the GNPS molecular networks. The t-SNE eases the interpretation of the molecular network by clustering together compounds that show high cosine scores, which eases the interpretation of the molecular network (Olivon et al., 2018).

There are different metrics to calculate spectral similarity. Indeed, cosine and modified cosine score might not often be the optimal choice for the construction of similarity networks. For example, compounds that show the same fragmentation pattern (i.e., the same neutral loss) but differ in the observed m/z show low cosine scores. It has been shown that Spec2Vec, a recently developed Python package that calculates spectral similarities based on fragmental relationships between large datasets, shows better overall performance than cosine-based scores, which were originally developed for matching fragmentation-rich electron ionization (EI) spectra (Huber et al., 2021).

Another approach to estimate spectral similarities is the use of hypothetical neutral loss spectra. An algorithm called core structure-based search (CSS) has been developed to calculate the spectral similarity between the mass difference between pairs of fragments ions. The CSS algorithm showed good performance in finding structurally relevant similarities (Xing et al., 2020). MS^2^ data and its analysis are crucial for accessing the chemical structure of unknown metabolites. It has been shown that the combination of different bioinformatic tools further enhances annotation success, which is of great importance, especially in untargeted metabolomics (Schmid et al., 2021).

### Correlation and Association Networks

Metabolites that are connected in metabolic pathways often show co-dependency, which can be seen by their orchestrated concentration (i.e., abundance) changes. So, the metabolites’ concentrations are correlated between metabolites that are associated or co-regulated within metabolic pathways (Rosato et al., 2018). Correlations of untargeted LC-MS metabolomics data are calculated by pairwise comparison of the peak intensity of all features, which results in a correlation adjacency matrix. In a correlation network, two metabolites are linked if their correlation value reaches a given (user-defined) threshold, which is considered as a significant correlation level (Table 4).

**TABLE 4:** Description of the key characteristics of correlation and association networks.

Correlation and association networks’ main characteristics	
Nodes	Features (intensity), low-level annotations (m/z)
Edges	Correlation between the abundances of two metabolites
Main Hypothesis	Metabolic processes imply metabolites’ abundance that depends on other metabolites’ abundance. Thus, metabolites with correlated abundance might be metabolically related
Limitations	The correlation or association between two metabolites (or features) does not systematically represent a metabolic relationship (e.g., substrate-product) or interdependence (e.g., co-regulation). Thus, correlation does not necessarily imply causal, biological, or chemical relationships

Most commonly, Pearson correlation is used to calculate correlations. However, due to tight metabolic control and the presence of long reaction sequences, standard Pearson correlation typically yields to highly connected and dense networks, which are hard to analyze and interpret. Gaussian graphical modeling uses partial instead of full correlation, and corrects for indirect correlation (i.e., when two metabolites are correlated just because they are both correlated with a third one). Therefore, using Gaussian graph modeling, only direct correlations can be found, which in turn allows us to construct meaningful networks containing potential direct reaction partners (Krumsiek et al., 2011). Benedetti et al. further compared the networks obtained using Pearson correlation, exact partial correlation, and partial correlation determined by GeneNet (Benedetti et al., 2020). They observed a dense network with an increased number of edges at increasing sample size for the Pearson correlation, whereas the partial correlation network (established with GeneNet) remained more stable. Furthermore, the statistical cut-off filter used to define the correlation threshold was more stable at varying the sample size using GeneNet than Pearson or partial correlations.

Another approach to statistically create metabolic networks is the weighted correlation network analysis, also known as weighted gene co-expression network analysis (WGCNA). In contrast to canonical correlation network analysis, the edges (which represent the correlation coefficients between features) are weighted by an exponent, such that the distribution of the weighted coefficients follows a power-law distribution, i.e., WGCNA assumes a priori a scale-free topology of the underlying network (Zhang and Horvath, 2005; Langfelder and Horvath, 2008). Nevertheless, to the best of our knowledge, it has not been proved yet if the statistical associations of the metabolites (or the subset acquired by GC- and LC-MS-based technologies) underlie such a scale-free topology.

WGCNA was originally applied to transcriptomics data, but it has also been recently employed for network generation using metabolomics data from human and human microbiome (Osterhoff et al., 2014; Pedersen and Sofia, 2018; Vernocchi et al., 2020; Murga-Garrido et al., 2021; Petersen et al., 2021), animal (Wu et al., 2021), and plants (DiLeo et al., 2011). (Samal and Martin, 2011).

## Knowledge Representation as Networks

### Genome-Scale Metabolic Networks and Graphs

**FIGURE 3:**
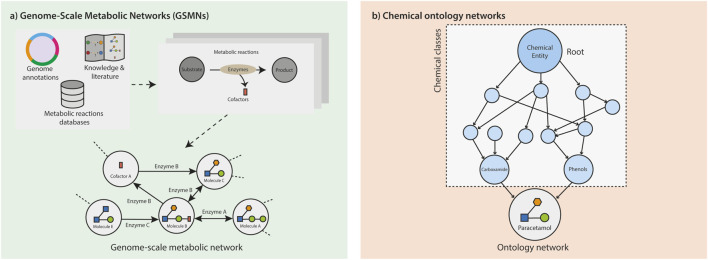
Representation of knowledge as networks. **(A)** Genome-scale metabolic networks: reconstructed from different sources of knowledge, such as from the enzymes identified in the annotated genome of the organism under study, the metabolic reactions databases, and/or biochemical knowledge and literature. The known metabolic reactions in an organism are the basis to generate a genome-scale metabolic network, where the metabolites are represented as nodes that are linked by (directed or undirected) edges, which represent the reactions converting the metabolites. **(B)** Chemical ontology networks: structure of relationships represented as a semantic network, where the nodes represent chemicals or chemical classes as “concepts”, bearing all their properties and definition, and that are connected by class membership.

Genome-Scale Metabolic Networks (GSMNs) are based on the current knowledge of the metabolism of a given organism (e.g., human metabolic network Human 1 with 13,417 reactions and 4,164 metabolites) (Robinson et al., 2020). They are usually drafted from genome annotations and reaction databases, before manual curation by domain experts, using available literature and simulation results (Table 5). They encompass the gene–reaction–metabolite information with the matrix associating metabolites to reactions, and the association of reactions to their corresponding genes and enzymes (Thiele and Palsson, 2010) (Figure 3A). GSMNs are frequently used to simulate metabolic fluxes via constrained-based metabolic modeling (Becker et al., 2007; O’Brien et al., 2015). Nonetheless, we will focus here on the use of GSMNs as graphs, which we will refer to as Genome-Scale Metabolic Graphs (GSMGs). Different graphs (directed or undirected) can be derived from GSMNs (Lacroix et al., 2008). For instance, reaction graphs represent the reactions as nodes, and two reactions are connected by an edge if the product of the first reaction is the substrate of the second one. On the other hand, the nodes of a compound graph represent metabolites that are connected by edges if they are substrates and products of the same biochemical transformation. Graph-based analysis methods can be applied to GSMGs to study both the metabolism and metabolomics data (Lacroix et al., 2008; Cottret and Jourdan, 2010; Frainay and Jourdan, 2017). For instance, path searches in GSMGs have been used to infer metabolic pathways connecting metabolites of interest. While supplanted by flux methods for such goals, path searches are still used for metabolomics data clustering and visualization (Liggi and Griffin, 2017; del Mar Amador et al., 2018). While GSMG analysis has been mainly focused on path search, graph theory encompasses a vast range of applications. Centrality analysis, for example, aiming at identifying key nodes in a graph, is quite popular for regulation and protein interaction network analysis, and has been applied a few times to metabolic networks as well (Faust et al., 2010; Bánky et al., 2013; Frainay et al., 2019). Beyond metabolomics data analysis, graph-based metrics have been used more to characterize and compare whole metabolic networks (Ma and Zeng, 2003; Mazurie et al., 2010).

**TABLE 5:** Description of the key characteristics of genome-scale metabolic networks and graphs.

Main characteristics of genome-scale metabolic networks and graphs	
Nodes	A “pool” of compounds (not restricted to small molecules)
Edges	Substrate-product relationships from known reactions
Main Hypothesis	Using genome annotation, reaction databases, and manual/semi-auto curation to generate a model of an organism’s metabolism
Limitations	There are gaps in the knowledge or predicted metabolic reactions in organisms, which creates an incomplete network of the metabolism

It is important to note that GSMNs do not cover all the metabolic products identified by metabolomics analysis, suggesting the absence of metabolic reactions and metabolites in the networks, as previously shown with the human GSMN (Frainay et al., 2018). This is a well-known problem as the GSMNs are biased by a reconstruction based on available genome annotations and knowledge of enzymatic reactions (Thiele et al., 2014; Pan and Reed, 2018). In consequence, gaps in the metabolic pathways are not always filled, as such gaps may also be due to enzymatic promiscuity and underground metabolisms (Notebaart et al., 2014; Pan and Reed, 2018).

The format in which GSMNs are stored can impact the graph structure and therefore the analysis of the graph, which is inconvenient. GSMNs are mainly shared in SBML format, which is an exchange format for computational models (not restricted to biochemical reactions) in biology (Hucka et al., 2003). SBML is mainly oriented towards quantitative models, which is why it has become the main support for GSMNs, given the popularity of GSMNs application for flux analysis. Building a network from a file in SBML format implies that the nodes correspond to a particular “species”. It should be noted that the species nodes can represent other biological entities than metabolites (such as proteins, generic degradation products or even the whole “biomass”). Furthermore, due to the GSMNs being tailored for flux modeling, the species actually represent pools of available biological entities at a given time and location. Consequently, SBML tends to represent the same metabolite as multiple species (“pool”) in different compartments, with a specific quantity that will be used for flux simulations. While SBML standard allows linking the species describing the same metabolites since version 2, in practice, those links are rarely defined. This leads to “duplicated” compartment-specific metabolites in many GSMNs, which differ from experimental networks in general, as compartment location is rarely available for metabolomics data. An alternative to SBML to represent metabolism knowledge is the BioPAX standard (Demir et al., 2010), oriented towards a semantic description of biological processes for indexing, sharing, and integration purposes, rather than quantitative modeling (Strömbäck and Lambrix, 2005). A network built from a BioPAX standard will have nodes that correspond to resources that describe biological entities, which are described using ontology vocabulary and linked to multiple information. However, BioPAX standard is mainly used at the individual pathway-level rather than the genome-scale level. Both exchange formats (SBML and BioPAX) represent knowledge about metabolism through lists of biochemical reactions, referencing metabolites as substrates or products (Strömbäck and Lambrix, 2005). A direct network translation would lead to a “bipartite metabolic graph”, where both reactions and compounds are explicitly represented as nodes. Compounds are thus never directly connected by an edge, but always through a reaction node, which differs from the structure of experimental networks, where related compounds are directly linked by edges.

### Chemical Ontology Networks

Chemical ontologies aim at providing a structured and formalized representation of chemical concepts. By describing an explicit structure of relationships among compounds, it can easily be represented as a semantic network that can be processed (Table 6).

**TABLE 6:** Description of the key characteristics of knowledge networks and graphs.

Main characteristics of knowledge networks and graphs	
Nodes	Chemical compounds and chemical classes
Edges	Class membership (subsumption) and other optional semantic relations
Main Hypothesis	Knowledge can be organized and represented as a network/graph by manual curation from domain experts and semi-automatic class assignments
Limitations	Ontologies representing the same things might still differ and can be mapped to different concepts

One of the main differences with the other presented networks is that, in chemical ontologies, the links do not represent (or suggest) biochemical/metabolic relationships that involve the transformation of one node into another. Rather, they represent subsumption relations between chemical compounds and broader chemical classes. For example, the ChEBI ontology links the node “paracetamol” to “carboxamide” and “phenols”, and each class back to higher classes, such as organic aromatic compounds (see Figure 3B). These graphs are directed acyclic graphs since they are organized hierarchically, are directed, and do not contain cycles. Importantly, in an ontology, molecules can belong to multiple parent classes. The compounds typically found in experimental networks lie as terminal nodes, and the rest of the nodes represent chemical classes. It is also mostly the case for GSMNs, but it is not rare to find nodes corresponding to classes (e.g., “a fatty acid”) (Poupin et al., 2020). Chemical ontologies can also integrate other kinds of relationships directly linking molecules, such as tautomers or conjugates (which can create cycles in the networks). The ChEBI ontology also links chemical compounds and classes to other concepts: their chemical/biological “roles” (e.g., emulsifier or neurotransmitter) (Degtyarenko et al., 2007). It is important to note that the class hierarchy of chemical ontologies is built manually by domain expert consortia, and the annotation of chemical instances to classes is either done manually or automatically if a class definition can be expressed as a set of formal rules.

Graphs built from ontologies allow detecting related compounds through their belonging to a shared class. Moreover, beyond finding “sibling” compounds, a graph distance between terminal nodes through their most precise common class can be computed to quantify relatedness between any pair of compounds. Such distances based on the ontology’s graph structure are a common form of semantic similarity, which found many applications in functional ontologies, such as the Gene Ontology (GO) (Ashburner et al., 2000).

Some specific tools allow fetching the chemical classification of a compound, which can then be used to generate the chemical ontology network of each compound. For example, ClassyFire allows to automatically assign chemical classification based on the compound’s structure (e.g., SMILES), using the ChemOnt ontology (Feldman et al., 2005; Djoumbou Feunang et al., 2016). Another tool, CANOPUS, can predict the chemical class based on MS^2^ data using ClassyFire and the ChemOnt ontology (Dührkop et al., 2020).

## Combining Networks Analysis and Multi-Layer Networks

Each of the previously presented networks (both knowledge-based and experimental) represents a different aspect of metabolism. The combination of two or more of such networks brings more comprehensive and informative analysis than a single network, by bringing different angles to the data and combining specific advantages of each network.

For instance, to improve annotations of metabolite features, spectral similarity networks can be combined with different information, such as chemical ontologies or mass difference networks. ChemRICH, for example, is a chemical similarity enrichment analysis that uses Tanimoto chemical similarity and ontologies to associate the metabolic structures from the similarity network with possible metabolic classes in the ontologies network (Barupal and Oliver, 2017). The main benefit of ChemRICH, as compared to classical pathway mapping, is a higher coverage because missing compounds in chemical ontologies can be mapped. Another tool developed to improve metabolite annotation is MolNetEnhancer, which combines molecular networks with chemical ontologies generated by ClassyFire and results from diverse in-silico annotation tools (Ernst et al., 2019). MolNetEnhancer shows great improvement in annotations, even without a prior library match in GNPS. FT-BLAST is a tool that uses fragmentation trees and their comparison to compounds in databases to annotate unknown compounds.

A fragmentation tree illustrates the fragmentation pattern of a compound by representing the molecular formulae of the fragments as nodes, and the neutral losses as edges (Rasche et al., 2012). Note that the in-silico annotation tool CSI:FingerID is also based on fragmentation trees (Dührkop et al., 2015). Moreover, iMet deals with the issue of metabolite annotations that were not present in any database. It uses the spectral similarity and the mass difference of the unknown compounds, and the metabolites present in the databases, in order to find putative neighbor metabolites that show high similarity and that are connected by chemical transformations (Aguilar-Mogas et al., 2017). This way, mass difference networks can be greatly enhanced by combining them with other approaches, such as correlation or spectral similarity networks (Aguilar-Mogas et al., 2017).

To further improve annotation, correlations between the concentration (i.e., abundance) of metabolites that are spectrally similar can be included to analyze metabolomics data. Indeed, it is very likely that, besides having a high spectral similarity, the concentration of metabolites that are connected via biochemical reactions also have a high correlation. Gaquerel et al. utilize in-source fragmentation patterns and correlation networks to improve MetFrag annotation results (Gaquerel et al., 2013). The combination of correlation networks with other metabolic networks can bring new insights into the metabolomics data. For example, Quell et al. demonstrated the potential of combining correlation networks (using Gaussian graphical modeling) with GSMNs and metabolite-gene association networks (derived from genome-wide association studies) to identify unknown metabolites from cohort studies (Quell et al., 2017). However, correlations and associations, in general, emerge due to different mechanisms, so the interpretation is not always straightforward (Steuer, 2006). For example, many associations between metabolite levels (e.g., strong correlations) do not happen between metabolites that are neighbors in the GSMN or that are directly involved in the same metabolic pathways. Analyzing and interpreting the association and correlation networks alongside complementary networks, such as GSMNs, help reduce spurious associations by using the biological knowledge incorporated in GSMNs (Benedetti et al., 2020).

GSMNs can help annotate untargeted metabolomics datasets, as the metabolites and their relationships via metabolic reactions can be analyzed to enhance metabolites’ annotations based on the biochemical context (Silva et al., 2014). First, metabolites (and potentially their structures) present in a GSMN represent a knowledge base of the metabolome/lipidome of a given organism. It must be noted that, in the past, GSMNs often lacked detailed structural curation and chemical identifiers, and metabolite names are often rather arbitrary. However, different improvements were suggested and are slowly adopted by the GSMN community (Witting, 2020).

Here, untargeted metabolomics data could be used to help to improve the GSMNs by identifying missing metabolites and filling missing metabolic pathways. For example, metabolites predicted from the WormJam GSMN have been compared against detected metabolites in the nematode Caenorhabditis elegans (C. elegans) in different studies (Salzer and Witting, 2021). Interestingly, the overlap of detected and predicted metabolites was rather modest (less than 40%). Plenty of metabolites beyond the consensus model were found, and structural similarity (based on chemical similarity using Tanimoto distances) has been suggested as an option to identify structurally related molecules (Witting et al., 2018).

Combining experimental network methods with biochemical knowledge-based networks can open new avenues. For example, the recently published tool LINEX allows to analyze lipidomics data by combining lipid metabolic reactions networks analysis with correlation networks (Köhler et al., 2021). With this method, Kohler et al. interpreted the lipidomics correlation networks in the context of biochemical reactions and found new insights on lipid metabolism in three previously published datasets (Köhler et al., 2021). In the same context, MetDNA uses MS/MS similarity networks and metabolic reaction networks. When two metabolites are connected by a reaction in the metabolic reaction network (i.e., when they are neighbor nodes), it is likely that they also show high similarity in the MS/MS similarity network, which can be used to weight their annotation confidence (Shen et al., 2019). By providing a controlled vocabulary, chemical ontologies, such as ChEBI or ChemOnt (Feldman et al., 2005; Degtyarenko et al., 2007), also contribute to the ease of interoperability between networks and data, notably by being frequently referenced in GSMNs and used in many chemical libraries. The controlled vocabulary combined with the distances between the nodes in the ontology offer a useful opportunity for handling partial identification of metabolites in metabolomics data (e.g., in case of lipids (PC(32:1)), since they allow to map such data onto metabolic pathways, using ontology from one specific compound (as identified in the data) to a more generic class (as annotated in the network) (Poupin et al., 2020).

**FIGURE 4:**
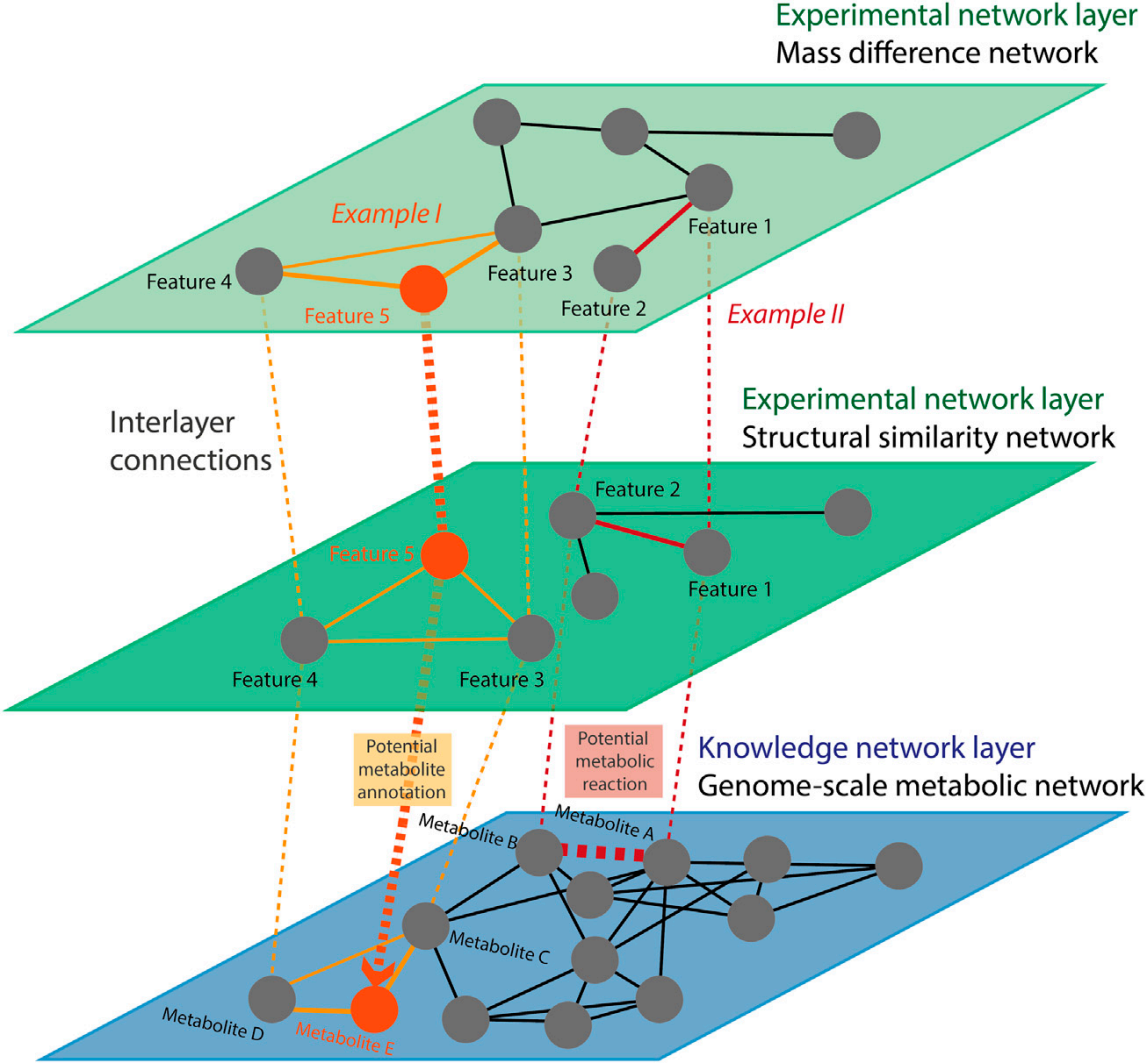
Multi-layer networks principle. Every network (either knowledge-based or experimental) is an independent layer. Common nodes (i.e., identified metabolites) are connected to themselves across the different layers by inter-layer edges. The set of nodes is common in the experimental layers, but we omitted some nodes for the sake of simplicity. The edges of the individual layers and between them can be used, for example, to identify potential metabolite annotations (Example I) and metabolic reactions (Example II). Multi-layer networks allow preserving the topology and organization of each individual network. In Example I, features 3 and 4 were identified as metabolites C and D, respectively. In both experimental layers, these two features are connected with each other and with feature 5. Similarly, in the knowledge-based layer, metabolites C and D are connected with each other and with metabolite E. Therefore, it is likely that feature 5 corresponds to metabolite E. In the same way, features 1 and 2, identified as metabolites A and B, respectively, are connected to each other in the experimental layers but not in the knowledge-based one. In Example II, the metabolite A and B are separated by a mass difference corresponding to known biotransformation (e.g., a phosphatase as in Figure 2A) in the layer 1 and are connected by a high structural similarity in layer 2. This represents a potential novel metabolic reaction occurring between metabolites A and B in layer 3.

Another approach to combine networks and analyze them could be to construct multi-layer networks. Multi-layer networks are particularly interesting as they allow viewing the metabolism from different but complementary perspectives (one per layer) while keeping the individual features (such as the topology) of each layer (Figure 4). Multi-layer networks are a useful approach to bring together multiple networks and interlink information across network types, for example between experimental and knowledge networks. As shown in Figure 4, the links between identified metabolites (i.e., nodes with interlayer edges between the experimental layers and the knowledge-based layer, represented as dotted lines) can be used to identify unknown features (Figure 4, Example I) or to identify a potential novel metabolic reaction (Figure 4, Example II). Multi-layer networks methods are already applied to multi-omics data (Hammoud and Kramer, 2020; Malek et al., 2020), but would benefit metabolomics data analysis by integrating metabolomics experimental and knowledge-based networks.

## Conclusions and Future Directions

Fundamentally, metabolites are the small molecules that are the components of the metabolism. Metabolites are consumed or produced via metabolic reactions mostly driven by biomolecules, such as proteins and genes. In order to study the metabolism and to have a global overview, we can represent the reactions as a network. Current knowledge of metabolism and chemical compounds can be used to generate genome-scale metabolic networks (Figure 3A) and ontology-based networks (Figure 3B), respectively.

In addition, we can generate other types of networks using experimental data. Indeed, metabolomics data capture different aspects and properties of the chemical compounds that constitute the metabolism. In this review, we described the most common networks that can be built based on the interactions and relationships between the measured compounds. We divided the experimental networks into four types: mass difference networks (Figure 2A), adduct and feature networks (Figure 2B), structure and MS/MS similarity networks (Figure 2C), and correlation networks (Figure 2D). The capabilities of those networks to represent the relationships between components are used to annotate and identify metabolites in untargeted MS-based metabolomics data.

In the end, each of the networks described here is useful for specific aspects of metabolomics data analysis and/or interpretation, but they also have limitations. Hence, integrating different networks into multi-layer networks holds great promise to combine all the information and derive new biological insights (Figure 4). Particularly, the combination of knowledge-based networks with experimental networks would help to use prior metabolic or chemical knowledge to improve the metabolites’ identification and interpretation in biologically relevant contexts.

In the future, with improved metabolite coverage, annotation, and identification, the combination of networks will enable new data analytical approaches. We therefore think that the development of approaches and algorithms for the analysis of metabolomics multi-layer networks will be at the center stage and will gain more and more attention. The multi-layer networks’ approach goes beyond mere metabolomics data and will allow integrating multiple omic data (as independent layers), including metabolomics. This will finally enable the analysis of metabolism with a systems biology approach.
